# Clinical exome sequencing elucidates underlying cause of death in sudden unexpected death of infants: two case reports

**DOI:** 10.1007/s00414-023-03065-3

**Published:** 2023-07-24

**Authors:** Laura Jane Heathfield, Lorna Jean Martin, Yolande van der Heyde, Itumeleng Molefe, Raj Ramesar

**Affiliations:** 1 Division of Forensic Medicine and Toxicology, Department of Pathology, Faculty of Health Sciences, University of Cape Town, Cape Town, South Africa; 2 MRC/UCT Research Unit for Genomic and Precision Medicine, Division of Human Genetics, Institute of Infectious Diseases and Molecular Medicine, Department of Pathology, University of Cape Town, Cape Town, South Africa

**Keywords:** Sudden unexpected death in infants (SUDI), Molecular autopsy, Bronchopulmonary dysplasia, Mannose-binding lectin deficiency, Polygenic mechanism, Postmortem forensic genetics

## Abstract

Sudden unexpected death in infants (SUDI) is a traumatic event for families, and unfortunately its occurrence remains high in many parts of the world. Whilst cause of death is resolved for most cases, others remain undetermined following postmortem investigations. There has been a recognition of the role of genetic testing in unexplained cases, where previous studies have demonstrated the resolution of cases through DNA analyses. Here we present two case reports of SUDI cases admitted to Salt River Mortuary, South Africa, and show that underlying causes of death were determined for both infants using clinical exome sequencing. The first infant was heterozygous for a variant (rs148175795) in *COL6A3*, which suggested a bronchopulmonary dysplasia phenotype. This hypothesis led to finding of a second candidate variant in *DMP1* (rs142880465), which may contribute towards a digenic/polygenic mechanism of a more severe phenotype. Histological analysis of retained tissue sections showed an asphyxial mechanism of death, where bronchiolar muscle weakness from an underlying bronchopulmonary dysplasia may have contributed to the asphyxia by affecting respiration. In the second infant, a homozygous variant (rs201340753) was identified in *MASP1*, which was heterozygous in each parent, highlighting the value of including parental DNA in genetic studies. Whilst mannose-binding lectin deficiency could not be assessed, it is plausible that this variant may have acted in combination with other risk factors within the triple-risk model to result in sudden death. These results may have genetic implications for family members, and represent possible new candidate variants for molecular autopsies.

## Introduction

Sudden unexpected death in infants (SUDI) is when an infant less than 1 year old dies so rapidly from the onset of symptoms that the cause of death cannot be confidently diagnosed by the infant’s doctor [1, 2]. SUDI cases form a substantial portion of the already high caseload experienced in forensic mortuaries globally, and in South Africa [3]. Whilst most SUDI cases are diagnosed with causes of death following postmortem investigation, a subset of cases remains unexplained. These cases may be diagnosed as sudden infant death syndrome (SIDS), if the cause of death remains undetermined following death scene investigation, clinical history review, and a full autopsy [2]. At South African forensic mortuaries, unexplained SUDI cases also include those which do not meet the criteria for SIDS, or subcategories thereof, owing to the resource-limited setting and inability to carry out core ancillary investigations in all cases [3].

Since SIDS is the leading cause of post-neonatal and infant death in developed countries [4], there has been much effort to further elucidate underlying causes. One of these has been the recognition of genetic variants contributing towards death [5]. This prompted a research study at the University of Cape Town, which is linked with Salt River Mortuary in South Africa, to prospectively investigate genetic variants in South African SUDI cases. To date, over 250 SUDI cases have been recruited, where the majority have undergone targeted genetic screening [6] and a subset have been sequenced for a panel of 43 genes that are associated with cardiac arrhythmias [7]. Two of the infants which had negative results in previous analyses, and for whom parental samples were available for genetic analysis, were prioritised for further genetic testing. The results of these analyses are presented as case reports here.

## Case histories

The first infant was an 8 week old South African female of Mixed Ancestry. She was born premature at 32 weeks via an emergency caesarean section, with a low birth mass of 2135 g. The mother received iron supplements during pregnancy and occasionally smoked. She reported that she did not drink alcohol or use drugs. The baby was exclusively breastfed and was reported to be healthy. The baby lived in a ‘Wendy house’ (wooden shed) with her parents and two siblings, aged 1 and 2 years old, respectively, at the time of the demise of the proband. All five family members co-slept on one large adult bed (foam rubber mattress), with the infant usually sleeping on her side without a pillow. Co-sleeping with multiple family members is common practice within this community [8].

One night, the mother woke up and felt that her baby was cold. The baby was found on her back with her face up and no items covering her face. The mother reached to put the infant on her chest to keep her warm, when she realised that the baby was unresponsive. According to the history given by the mother, the baby had a “mild” blocked nose.

The second case was also a female South African infant of Mixed Ancestry and was 2 months old at the time of her demise. She was born premature, at 37 weeks gestation, via natural delivery with a low birth mass of 2330 g. The mother received antenatal care but self-reported smoking 3–5 cigarettes per day during pregnancy. At the time of the interview with Forensic Pathology Services, she acknowledged that she knew smoking harms unborn babies, and she denied drinking alcohol and using drugs.

The baby received kangaroo mother care and was fed a combination of breastmilk and formula. The infant was fully up to date with vaccinations and was apparently well. On the night of her demise, the baby was put to sleep (co-slept with parents), and in the early hours of the morning, the mother woke up to her baby coughing blood. The baby was on her side, with one eye open and one eye closed. The mother described that she saw the baby turn blue as she rushed her to their neighbour for help.

## Postmortem examinations

The infants were admitted to Salt River Mortuary as SUDI cases for determination of cause of death. A death scene investigation was performed in both cases by Forensic Pathology Officers and full clinical history was available for both infants.

At autopsy, the first infant had no external signs of injury, and had a mass of 2500 g, length of 49 cm, crown rump length of 34 cm, and head circumference of 33 cm. A full-body X-ray was first performed using the Lodox® Xmplar-dr which showed no evidence of bone fractures or deformities. Examination of the head showed normal fontanelles and head shape, with no evidence of hygroma, encephalocoele, or diencephalic syndrome. The eyes, ears, and nose appeared anatomically normal, and blood was noted in the left nostril. A well-defined philtrum was present; there was no evidence of cleft lip, cleft palate, micrognathia, or microstomia. The lips and gingiva were blue in colour. The arms, legs, hands, feet, chest, abdomen, genitalia, and anus were all normal in structure. There was no evidence of spina bifida.

Internal organs were dissected, weighed, and examined, and were anatomically within normal range, except for the thymus which was enlarged (15.5 g; normal mass = 6.6 g). The lungs were firm and on cut section showed congestion and oedema. Notably, the heart was anatomically normal containing a closed foramen ovale and ductus arteriosus. The mural endocardium and valves were normal, and the coronary ostia and the coronary sinus were in normal position. The adrenal glands showed no surrounding haemorrhage and were of normal size. The cut surfaces revealed well-demarcated peripheral and central zones. Although the central zone was congested, there was no evidence of overt haemorrhage.

Overall, there was no evidence of injury to the body and there were no remarkable signs of disease. Tissue sections were retained for future histological examination, only if ancillary tests indicated this would be necessary. Various samples were obtained for biochemistry, virology, microbiology, and toxicology, all of which had unremarkable findings. The preliminary postmortem report indicated that the infant demised from natural causes.

The second infant had a mass of 3237 g, a length of 47.2 cm, a crown rump length of 33.1 cm, and a head circumference of 35.4 cm. The disparity between the crown rump length and the head circumference indicated imperfect growth. There were no external signs of trauma to the body and no congenital abnormalities. There were no external signs of dehydration or jaundice, and the skin showed no lesions. The Lodox® Xmplar-dr scan showed consolidation of the right lung in keeping with lower respiratory tract infection. Since foul play was not suspected, an internal autopsy was not performed to investigate the cause of natural death further. This is in keeping with the National Health Act No. 61 of 2003 [9], where natural deaths are not obligated to be investigated by the Forensic Pathology Services for exact cause of death determination.

Whilst both infants had a low birth weight, which is a risk factor for SIDS [10], the associated immaturity was not deemed sufficient to be the cause of death in either infant. Following informed consent from both sets of parents, a 4 ml blood sample was collected from each infant at autopsy for the purposes of the research study. Later, blood samples from the parents were also collected for analysis. A full family history was also obtained from both families with the aid of a genetic counsellor. Ethics approval for this study was obtained by the Institutional Review Board (HREC: 445/2015).

## DNA analyses

Deoxyribonucleic acid (DNA) was extracted from the blood samples as previously described [6]. DNA was quantified using Qubit® fluorometry (Thermo Fisher Scientific, Waltham, USA) and then diluted to 5 ng/µl using a two-step approach. Clinical exome libraries (4 811 genes which are known to be associated with a broad range of clinical phenotypes) were prepared using the TruSight One Sequencing Panel Series (Illumina, Cambridge, UK) according to the manufacturer’s Reference Guide [11]. Each trio’s samples were prepared and sequenced together (i.e. two multiplexes of three samples each) to ensure adequate coverage.

The pooled library was quantified using Qubit® fluorometry and then diluted to 6 pM using pre-chilled HT1 buffer (Illumina, Cambridge, UK) in a three-step approach. This was spiked with 1% 20 pM PhiX control (Illumina, Cambridge, UK) and placed into the MiSeq™ Reagent Kit v3 600 cycle cartridge (Illumina, Cambridge, UK) for sequencing on the MiSeq™ FGx (Illumina, Cambridge, UK). Fastq files were converted into VCF files using the integrated bioinformatics tools and were uploaded onto Illumina BaseSpace Variant Interpreter (publicly accessible: https://basespace.illumina.com/dashboard; date accessed 26/11/2018). Variants were called using GAKT version 1.6 [12], annotated using BaseSpace Annotation Engine 1.6.2.0, and were filtered according to predicted pathogenicity and prioritised according to clinical significance using the criteria outlined by the American College of Medical Genetics and Genomics and the Association for Molecular Pathology [13].

Candidate variants were explored using ClinVar (publicly accessible: https://www.ncbi.nlm.nih.gov/clinvar/) and peer-reviewed literature to assess the functional significance of the variants within the case contexts. The infants’ DNA sequences were the focus of the analysis, whereas the sequences from the parents were used only to assess the presence of de novo variants (i.e. those which occur in the infant but neither of the parents) as well as to assess the segregation of variants found in the infants.

## Results and discussion

### Case 1

A total of 10 722 variants were identified in the first infant, of which three were prioritised for further investigation (Table 1). The prioritised variants were all heterozygous in the infant and in at least one of the parents (i.e. none was de novo). The prioritised variants had previously been reported in patients with diagnosed diseases. This does not imply that the variant was causative of the disease in the patient, but merely that the variant was observed in a patient diagnosed with a particular disease. The clinical significance of these three variants were classified as pathogenic (*n* = 1) and variant of unknown significance (VUS) (*n* = 2).

**Table 1:**
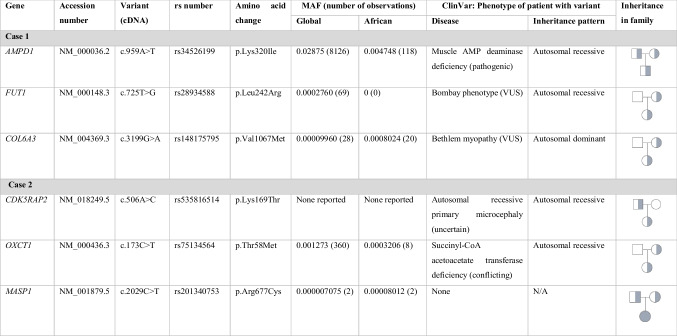
List of prioritised variants from clinical exome sequencing. Three variants in infant 1 and three variants in infant 2 were prioritised. They were all predicted to be pathogenic in silico, were rare, and had good quality scores from sequencing. All prioritised variants were missense variants; none were de novo. *MAF*: minor allele frequency; obtained from gnomAD (http://gnomad.broadinstitute.org; date accessed 26/11/2018)

The variant classified as pathogenic in *adenosine monophosphate deaminase* (*AMPD1*) (rs34526199) was associated with muscle AMP deaminase deficiency (MMDD) (ClinVar submission accession number SCV000109808.7). MMDD is an autosomal recessive disorder characterised by muscle weakening and exercise-induced muscle pain. Some homozygous individuals may be predisposed to heart disease [14] but many are asymptomatic [15]. The rest of the gene was analysed in the infant, which showed no variants, and this excluded the possibility of the infant being affected by a compound heterozygote mechanism [16]. However, since both parents were carriers, a sibling or a possible future baby will have a 25% chance of being homozygous for this variant and affected by MMDD. As such, the family will be referred for genetic counselling in this regard to inform them of this finding and to suggest screening of siblings for this variant.

A VUS (rs28934588) was found in *fucosyltransferase 1 (H blood group)* (*FUT1*). This gene is involved in the synthesis of the precursor of the H antigen in blood, and thus, mutations in this gene can cause the H-Bombay blood type by a homozygous or compound heterozygous mechanism. However, affected individuals are not affected unless given blood which is incompatible with their blood type [17]. There was no other variant identified in the same gene, and since this infant did not receive a blood transfusion, it was unlikely that this variant contributed towards death.

A second heterozygous VUS (rs148175795) was identified in *collagen type VI alpha 3 chain* (*COL6A3*), which has previously been identified in a patient with Bethlem myopathy 1 (BTHLM1) (ClinVar submission accession number SCV000820320.1). The *COL6A3* gene encodes the largest of the three alpha chains of collagen type VI which is an essential filament in most connective tissue. The amino terminal globular domain of the chain binds extracellular matrix protein, thereby providing vital structure and organisation of the cell components. Mutations in *COL6A3* have been associated with a range of muscular disorders with different degrees of severity including BTHLM1 and Ullrich congenital muscular dystrophy (CMD). BTHLM1 is an autosomal dominant disease, characterised by proximal muscle weakness and contractures, with early prenatal or neonatal onset. Ullrich CMD has an autosomal recessive inheritance pattern, and is a more severe form with later childhood onset, which can also affect respiratory function more critically.

In the clinical history, the mother did not report associated symptoms (e.g. hypotonia or torticollis), and no contractures were noted at autopsy in the case study, which suggests that BTHLM1 was unlikely. Further, the mother was also heterozygous for the same variant, but was asymptomatic. The involvement of the cardiovascular system in BTHLM1 has also been proposed, but a review showed that the heart is only mildly affected in the minority of patients and perhaps rather due to co-morbidities [18]. However, exome sequencing in a case–control study of neonates revealed 14 candidate genes that were significantly associated with bronchopulmonary dysplasia, one of which was *COL6A3* [19]. Bronchopulmonary dysplasia is a chronic lung disease, typically in premature infants, which manifests by respiratory impairment, and can result in respiratory arrest and death [20].

Gene ontology (GO) analysis showed that the candidate genes clustered in certain pathways, including collagen fibril organisation, morphogenesis of embryonic epithelium, and regulation of Wnt signalling [19]. Suboptimal functioning of these pathways have previously been proposed to predispose premature infants to develop bronchopulmonary dysplasia [21].

Li et al. (2015) hypothesised that since bronchopulmonary dysplasia does not follow typical Mendelian inheritance, and many affected patients were heterozygous for variants in the identified candidate genes, variant alleles with loss of function contribute towards the disease phenotype [19]. The authors went on to show that candidate genes in patients with bronchopulmonary dysplasia were indeed significantly more haploinsufficient compared to the other protein-coding genes in the genome, which was not the circumstance in controls [19]. Analysis of gene expression in pulmonary tissue of patients showed an upregulation of candidate genes, and functional analysis in rates exposed to a hyperoxic environment has shown elevated expression of the candidate genes in comparison to controls [19]. Lastly, a significant association was found between infants with bronchopulmonary dysplasia and SIDS in 1982, where the incidence of sudden death was seven times greater in patients than controls [22].

These findings therefore suggest evidence that the SUDI case admitted to Salt River Mortuary, with the heterozygous variant rs148175795 in *COL6A3*, may have been predisposed to an undiagnosed bronchopulmonary dysplasia. This is supported by the autopsy findings of blue lips, blood in the nose and firm lungs, which are all consistent with a hypoxic event. To further investigate this hypothesis, the other 13 candidate genes that were significantly associated with bronchopulmonary dysplasia in the Li et al. (2015) study were assessed in the infant. A heterozygous variant in *dentin matrix acidic phosphoprotein 1* (*DMP1*) (rs142880465), which was also predicted to be pathogenic in silico, was rare (MAF = 0.000289; gnomAD) and was inherited from the father. Given the dosage sensitivity model for bronchopulmonary dysplasia, the two heterozygous variants present in two candidate genes (one inherited from each parent) in a premature infant provide support for the hypothesis of respiratory arrest as a mechanism for the sudden death of the infant.

Histological examinations of tissue sections retained at autopsy were assessed and the main findings were as follows:

- The thymus showed very short duration stress reaction (estimated to be 12–48 h in duration).
- The brain showed hypoxic neuronal injury.
- The lungs showed intra-alveolar haemorrhage, intra-alveolar septal haemorrhage, and haemorrhage within bronchi, which is inconsistent with bronchopulmonary dysplasia.

No fibrin thrombi were present in any of the sections to suggest bleeding disorder. Other causes for intra-alveolar haemorrhage such as sepsis and mitral valve stenosis were excluded. This is in keeping with no history of a bleeding disorder. The kidney and cerebellum maturation were appropriate for chronological age.

**Fig. 1:**
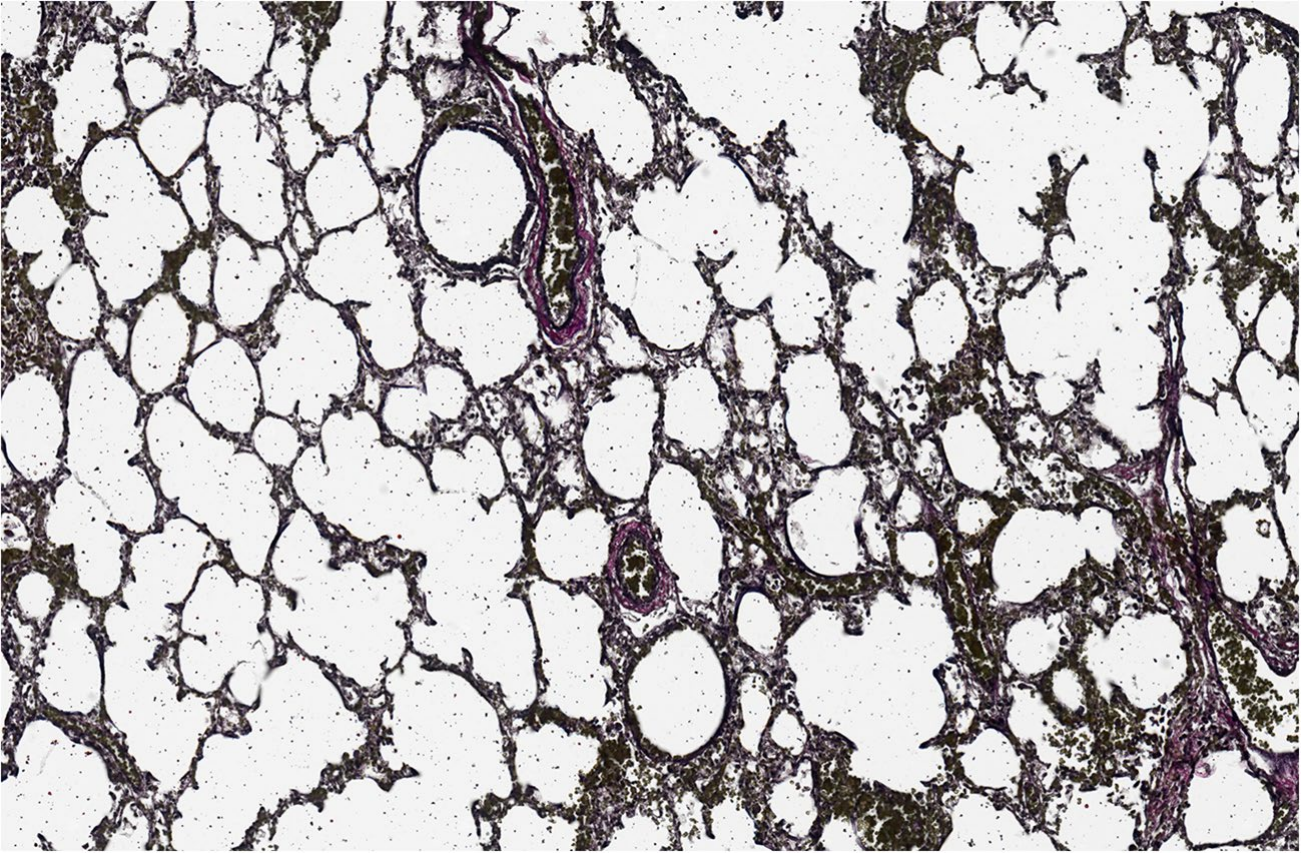
Elastic Van Gieson stain of lung tissue in case 1, showing no evidence of bronchopulmonary dysplasia

There was no evidence of bronchopulmonary dysplasia on histological examination of the lung tissue, as confirmed using the elastic Van Gieson stain (Fig. 1). The reason for this is most likely due to the baby not requiring mechanical ventilation and high oxygen concentrations. Dexamethasone (corticosteroid) was more than likely administered prior to the caesarean section being performed.

Based on all postmortem findings, the mechanism of death was considered to be asphyxial; however, the cause for this asphyxia could not be ascertained conclusively. Whilst the skeletal muscle histologically showed no abnormalities, muscle weakness (or some degree thereof) cannot be excluded in this case and may possibly have contributed to the asphyxia by affecting respiration. Upper airway obstruction due to the blocked nose may have also played a role, and given that co-sleeping occurred, the possibility of overlying cannot be excluded — although this was denied by the mother. Thus, whilst there was no histological evidence of bronchopulmonary dysplasia, this may have been due to the post-partum care received. Given the genetic results, myopathy cannot be excluded as having a direct bearing on the asphyxial mechanism of death in this case. This illustrates the importance of genetic analyses and multi-disciplinary testing in SUDI cases.

### Case 2

The second infant had 11 230 variants and three variants were also prioritised for further investigation (Table 1). Similarly, none of the prioritised variants occurred de novo. The first was a heterozygous variant (rs535816514) in *CDK5 regulatory subunit associated protein 2* (*CDK5RAP2*) which has been observed in a patient with autosomal recessive primary microcephaly (MCPH). This condition is characterised by an abnormally small brain and head which also causes intellectual disability. The SUDI case was unlikely to have this condition as the clinical record documented a normal head circumference at birth and no signs of MCPH observed at birth or at autopsy. Further, the infant was heterozygous for this variant, with no other variants in the same gene; as such, she would not have been affected by this autosomal recessive condition.

A heterozygous variant (rs75134564) was identified in the infant, which was in the gene *3-oxoacid CoA-transferase 1* (*OXCT1*). The variant has previously been found in patients with succinyl-CoA acetoacetate transferase deficiency (SCOT), which is when the body cannot metabolise ketones effectively. This disorder presents with ketoacidotic attacks, with symptoms of lethargy, loss of appetite, and vomiting, which are aggravated by infections, fevers, and periods of fasting. It has an early onset, with many individuals experiencing their first attack within their first few days of life.

Although this variant observed in the SUDI case was predicted to be pathogenic in silico, the consensus on ClinVar from multiple entries was that this variant is benign on its own (publicly accessible: https://www.ncbi.nlm.nih.gov/clinvar/variation/242800/; date accessed 28/11/2018). Rather, it was found to contribute towards pathogenicity when in a compound heterozygous state with another variant rs267606930 (NM_000436.3:c.398 T > A; p.Val133Glu) in the same gene [23]. However, this SUDI case did not have this second variant, or any other variant in *OXCT1*, and therefore was unlikely to have been affected by SCOT.

Lastly, one homozygous variant (rs201340753) was found in the infant, which was heterozygous in both parents and which met the criteria for pathogenicity for prioritisation. This variant was in the *mannan binding lectin serine peptidase 1* (*MASP1*) gene which encodes a serine protease which contributes towards the activation of the lectin complement system [24]. Within the innate and adaptive immune response, complement activation is critical to identify and remove pathogens, as well as to promote inflammation. The mannose-binding lectin (MBL) protein functions within this pathway by binding to various clinically relevant pathogens [25] and it is thought to also be involved in coagulation [26]. Loss of function of this gene has shown to be inhibitory of the complement pathway [27].

Genetic variants have been associated with MBL deficiency in serum [28] and MBL deficiency has been associated with increased infections, particularly respiratory tract infections [29]. It has also been associated with recurrent pregnancy loss [30], which is interesting within this case study where the mother had a previous stillbirth, as miscarriages, stillbirths, and SUDI have many overlapping risk factors and it has been suggested that may form part of a continuum spectrum [31]. Kilpatrick et al. (1998) also showed that SIDS cases who were MBL deficient had significantly greater MBL values compared to controls and suggested that some SUDI cases are precipitated by bacterial infections [32].

Whilst it is no longer possible to test for MBL deficiency in the deceased infant due to unavailability of appropriate samples, it is plausible that the homozygous variant in *MASP1* may have contributed towards an MBL deficiency. Within the context of the triple-risk model for SIDS [33], the genetic variant may have contributed towards the predisposition or vulnerability component in the infant, and combined with the infant being in a critical stage of development (2 months old) and signs of respiratory tract infection (trigger), may act together to adequately explain a possible mechanism for death. This may have been further exacerbated by maternal smoking, low birth weight, and other risk factors that were present.

## Conclusion

Clinical exome sequencing was conducted in two probands of SUDI cases, alongside their biological parents, in attempts to better understand specific causes of death and underlying mechanisms for SUDI. The molecular autopsies elucidated two variants which may be worthwhile investigating in larger cohorts of SUDI with similar circumstances at death. The first had a heterozygous variant (rs148175795) in *COL6A3*, which suggested a bronchopulmonary dysplasia phenotype. This hypothesis led to finding of a second candidate variant in *DMP1* (rs142880465), which may contribute towards a digenic/polygenic mechanism of a more severe phenotype. This prompted a detailed histological analysis of the retained tissue which suggested an asphyxial mechanism of death. The genetic findings suggested myopathy, which may have been an underlying contributor towards the cause of death.

In the second infant, a homozygous variant (rs201340753) was identified in *MASP1*, which was heterozygous in each parent. Whilst MBL deficiency could not be assessed, it is plausible that this variant may have acted in combination with other risk factors within the triple-risk model to result in sudden death; however, this requires further research where appropriate samples are obtained at autopsy for immunological analysis.

Immunology tests are not a routine ancillary investigation in the forensic pathology setting in South Africa, and perhaps more attention should be given to this important component in future research and SUDI investigations. Whilst these genetic findings do not change the overall manner of death as being natural, they do provide valuable insight into two SUDI cases, which may have genetic importance for family members. To this end, genetic counsellors have been engaged to converse with these families and explain these findings within the contexts of their respective family histories.

Overall, these two case reports demonstrate the value of including parental samples in the genetic analysis, to assist with co-segregation analysis of recessive disorders as well as to assess polygenic conditions, where variants in different genes may each contribute towards an overall affected phenotype. These findings also represent possible new candidate variants to assess in the future — both functionally as well as in case–control SUDI cases, to better understand their pathogenicity and potential role. Sequencing of the whole genome, whole exome, and mitochondrial DNA may also elucidate other novel variants which may be clinically relevant. These emerging avenues of molecular research are important for gaining deeper insight into mechanisms of SUDI, to resolve and prevent SIDS cases, and ultimately reduce infant mortality.

## Data Availability

The data relevant to this article are included herein. The larger datasets generated during the study are not available as the participants did not consent to their clinical exomes being shared.

## References

[1] Mason J (1995). Forensic medicine for lawyers.

[2] Krous HF, Beckwith JB, Byard RW (2004). Sudden infant death syndrome and unclassified sudden infant deaths: a definitional and diagnostic approach. Pediatrics.

[3] du Toit-Prinsloo L, Dempers J, Verster J (2013). Toward a standardized investigation protocol in sudden unexpected deaths in infancy in South Africa: a multicenter study of medico-legal investigation procedures and outcomes. Forensic Sci Med Pathol.

[4] Hauck FR, Tanabe KO (2010). International trends in sudden infant death syndrome and other sudden unexpected deaths in infancy: need for better diagnostic standardization. Curr Pediatr Rev.

[5] Opdal SH, Rognum TO (2004). The sudden infant death syndrome gene: does it exist?. Pediatrics.

[6] Heathfield LJ, Bhengu W, Louw S (2020). Assessment of candidate variants causative of inborn metabolic diseases in SUDI cases in South Africa, and a case report. Int J Legal Med.

[7] Heathfield LJ, Watkins H, Martin LJ, Ramesar R (2021). Massively parallel sequencing of 43 arrhythmia genes in a selected SUDI cohort from Cape Town. J Pediatr Genet.

[8] Heathfield LJ, Martin LJ, Ramesar R (2020). A 5-year retrospective analysis of infant death at Salt River Mortuary, Cape Town. SAJCH South African J Child Heal.

[9] South African Government (2004) The National Health Act No 61 of 2003. Publicly accessible: https://www.gov.za/documents/national-health-act. Accessed 21 Jul 2023

[10] Mayor S (2016) Low birth weight is associated with increased deaths in infancy and adolescence, shows study. BMJ 353:i2682. 10.1136/bmj.i2682

[11] Illumina (2018) Trusight one sequencing panel series reference guide. Document #15046431 v03. 1–37. Publicly accessible: https://support.illumina.com/content/dam/illumina-support/documents/documentation/chemistry_documentation/trusight_one/trusight-one-sequencing-panel-reference-guide-15046431-03.pdf. Accessed 21 Jul 2023

[12] McKenna A, Hanna M, Banks E (2010). The Genome Analysis Toolkit: a MapReduce framework for analyzing next-generation DNA sequencing data. Genome Res.

[13] Richards S, Aziz N, Bale S (2015). Standards and guidelines for the interpretation of sequence variants: a joint consensus recommendation of the American College of Medical Genetics and Genomics and the Association for Molecular Pathology. Genet Med.

[14] Genetta T, Morisaki H, Morisaki T, Holmes EW (2001). A novel bipartite intronic splicing enhancer promotes the inclusion of a mini-exon in the AMP deaminase 1 gene. J Biol Chem.

[15] Verzijl HT, van Engelen BG, Luyten JA (1998). Genetic characteristics of myoadenylate deaminase deficiency. Ann Neurol.

[16] Abe M, Higuchi I, Morisaki H (2000). Myoadenylate deaminase deficiency with progressive muscle weakness and atrophy caused by new missense mutations in AMPD1 gene: case report in a Japanese patient. Neuromuscul Disord.

[17] Kelly RJ, Ernst LK, Larsen RD (1994). Molecular basis for H blood group deficiency in Bombay (Oh) and para-Bombay individuals. Proc Natl Acad Sci U S A.

[18] Finsterer J, Ramaciotti C, Wang CH (2010). Cardiac findings in congenital muscular dystrophies. Pediatrics.

[19] Li J, Yu KH, Oehlert J (2015). Exome sequencing of neonatal blood spots and the identification of genes implicated in bronchopulmonary dysplasia. Am J Respir Crit Care Med.

[20] Davidson L, Berkelhamer S (2017). Bronchopulmonary dysplasia: chronic lung disease of infancy and long-term pulmonary outcomes. J Clin Med.

[21] Baraldi E, Filippone M (2007). Chronic lung disease after premature birth. N Engl J Med.

[22] Werthammer J, Brown ER, Neff RK, Taeusch HW (1982). Sudden infant death syndrome in infants with bronchopulmonary dysplasia. Pediatrics.

[23] Song XQ, Fukao T, Watanabe H (1998). Succinyl-CoA:3-ketoacid CoA transferase (SCOT) deficiency: two pathogenic mutations, V133E and C456F, in Japanese siblings. Hum Mutat.

[24] Sato T, Endo Y, Matsushita M, Fujita T (1994). Molecular characterization of a novel serine protease involved in activation of the complement system by mannose-binding protein. Int Immunol.

[25] Neth O, Jack DL, Dodds AW (2000). Mannose-binding lectin binds to a range of clinically relevant microorganisms and promotes complement deposition. Infect Immun.

[26] Jenny L, Dobó J, Gál P, Schroeder V (2015). MASP-1 induced clotting—the first model of prothrombin activation by MASP-1. PLoS One.

[27] Skjoedt M-O, Hummelshoj T, Palarasah Y (2010). A novel mannose-binding lectin/ficolin-associated protein is highly expressed in heart and skeletal muscle tissues and inhibits complement activation. J Biol Chem.

[28] Aittoniemi J, Rintala E, Miettinen A, Soppi E (1997). Serum mannan-binding lectin (MBL) in patients with infection: clinical and laboratory correlates. APMIS.

[29] Aittoniemi J, Baer M, Soppi E (1998). Mannan binding lectin deficiency and concomitant immunodefects. Arch Dis Child.

[30] Christiansen OB, Kilpatrick DC, Souter V (1999). Mannan-binding lectin deficiency is associated with unexplained recurrent miscarriage. Scand J Immunol.

[31] Moscovis SM, Gordon AE, Al Madani OM (2015). Virus infections and sudden death in infancy : the role of interferon- γ. Front Immunol.

[32] Kilpatrick DC, James VS, Blackwell CC (1998). Mannan binding lectin and the sudden infant death syndrome. Forensic Sci Int.

[33] Filiano JJ, Kinney HC (1994). A perspective on neuropathologic findings in victims of the sudden infant death syndrome: the triple-risk model. Neonatology.

